# Association of circulating irisin levels with normal weight obesity, glycemic and lipid profile

**DOI:** 10.1186/s40200-016-0239-5

**Published:** 2016-06-27

**Authors:** Sarvenaz Mehrabian, Ehsaneh Taheri, Maryam Karkhaneh, Mostafa Qorbani, Saeed Hosseini

**Affiliations:** 1 Department of Clinical Nutrition, School of Nutritional Sciences and Dietetic, Tehran University of Medical Sciences, No 44, Hojjat-dost Alley, Naderi St., Keshavarz Blvd, Tehran, 1416-643931 Iran; 2 Endocrinology and Metabolism Research Center, Endocrinology and Metabolism Research Institute, Tehran University of Medical Sciences, Tehran, Iran; 3 Dietary Supplements and Probiotics Research Center, Alborz University of Medical Sciences, Karaj, Iran; 4 Chronic Diseases Research Center, Endocrinology and Metabolism Population Sciences Institute, Tehran University of Medical Sciences, Tehran, Iran; 5 Obesity and Eating Habits Research Center, Endocrinology and Metabolism Molecular -Cellular Sciences Institute, Tehran University of Medical Sciences, Tehran, Iran

**Keywords:** Irisin, Normal weight obesity, Glycemic and lipid profile

## Abstract

**Background:**

Irisin, a recently identified myokine/adipokine, has potential role in type 2 diabetes and obesity. Normal weight obesity (NWO) is associated with a significantly higher risk of developing metabolic syndrome and cardiometabolic dysfunction. The aim of this study was to investigate association of irisin level with NWO, glycemicand lipid profile in women.

**Method*s*:**

In this matchedcase-control study, 38 subjects with NWO (body mass index (BMI) <25 kg/m2 and BF % > 30) as case and 26 controls (BMI <25 kg/m2 and BF % < 30) were selected randomly from sport clubs in the East area of Tehran, Iran. In addition to anthropometric variables, including BMI and body composition, fasting blood sugar (FBS), fasting levels of irisin andinsulin, triglyceride (TG), total cholesterol (TC), high-density lipoprotein (HDL) and low-density lipoprotein (LDL) cholesterol were measured. All statistical analyses were performed with SPSS 18.0.

**Results:**

In univariate analysis, levels of irisin were significantly higher in subjects with NWO compared to controls (0.81 ± 0.41*vs.* 0.58 ± 0.26 ng/ml, *P* = 0.009). This association remained significant after adjusting for confounders (adjusted for energy intake, physical activity, waist circumference and BMI) (*P* = 0.049). In NWO, irisin level was not significantly correlated with all glycemic and lipid profile. In control group, only correlation ofirisin with insulin was statistically significant (*P* = 0.03).

**Conclusion:**

Serum irisin levels were higher in NWO subjects than controls. In control group, only the negative association between irisin and insulin levels was statistically significant. Further studies with larger sample size are clearly needed to evaluate the potential role of irisin in NWO subject and other disturbed metabolic conditions.

## Background

Normal weight obesity (NWO) is defined as a condition that is characterized by normal body mass index (BMI: 18.5–24.9 kg/m^2^) with increased percent body fat. Recent investigations have reported the association of NWO with cardiovascular risk factors, and also with increased mortality in women [1]. However, the exact prevalence of NWO in the general population is unknown.

The prevalence rates ofhigh waist circumference, waist to hip ratio, high serum levels of low-density lipoprotein (LDL) and triglycerides, low serum level of high-density lipoprotein (HDL), reduce insulin sensitivity (measured as HOMA-IR) and high C-reactive protein levels were higher in patients with NWO compared to subjects with normal weight and normal body fat mass [2].

Increasing evidence suggests that skeletal muscle is an endocrine organ, producing and releasing cytokines which have been termed “myokines” [3, 4]. Several of these myokines are involved in the regulation of physiological functions and metabolic pathways, including body metabolism and energy homeostasis [4, 5].

Irisin is a newly discovered plasma myokine/adipokine that is produced by the proteolytic cleavage of fibronectin type III domain containing 5 (FNDC5). In skeletal muscle, the peroxisome proliferator-activated receptor γ (PPAR- γ) coactivator 1α (PGC-1α) expression as a mediator of the effects of exercise, stimulates increased expression of FNDC5 as a precursor of irisin [6]. In addition to the myocytes, irisin is expressed in adipocytes, kidney, lungand liver tissues. However, the maximum expression of irisin was observed in skeletal muscles [7].

However, conflicting data on the role of irisin or FNDC5, as the precursor of irisin, in obesity and glucose metabolism have recently emerged. Some studies observed the increased blood concentration of irisin and FNDC5 expression in obese patients [8, 9]. In contrast, other studies have shown opposite results [7, 10]. While some researchers found the negative relationship between bloodirisin level and glycemic parameters [11], others reported that irisin levels positively correlated with the serum concentration of insulin, insulin resistance and fasting blood glucose [12]. In addition, irisin levels were significantly inversely associated with the onset of type 2 diabetes [13, 14] and with another pathway, such as inflammation and oxidative stress, which have previously been proposed to be involved in insulin resistance [15].

However, to our knowledge, no study to date has examined the relation between blood irisin levels in patients with the normal weight obesity. Therefore, we aimed to investigate association between irisin levelwithNWO, glycemic and lipid profile in women.

## Methods

### Subjects

This matched case–control study that performed from October 2014 to May 2015, women aged 19 to 39 years were selected randomly from sport clubs in the East area of Tehran, Iran. The study groups included 38 subjects with NWOand 26 healthy subjects as a control group. Two groups were frequency matched based on age groups. Normal weight obesity was defined as those subjects with a normal body mass index (BMI 18.5–24.9 kg/m^2^) and high body fat percentage (%BF) content (BF > 30 %). Subjects with normal BMI (18.5–24.9 kg/m^2^) and %BF content < 30 % of body weight were considered as controls. The age and BMI were matched between two groups.

Sample size was calculated based on a pilot study and according to two mean comparison formula .subject were selected by announcements in the youth sports club in Tehran among them, women aged 19–39 years with normal weight for height who had joined the club recently were selected to measure body composition using the BIA (bio electric impedance analyzer).

Subjects were excluded if they had a history of diabetes, liver and kidney dysfunction, hypertension, gastrointestinal, cardiovascular, thyroid and autoimmune diseases or diagnose infection. Pregnant and lactating individuals were not included andsubjects who used any drugs, supplements or diet for a long time and professional athletes were also excluded from the study.

### Ethics statement

This study was approved by the ethics committee of Endocrinology and Metabolism Clinical Sciences Institute, Tehran University of Medical Sciences (No: 1393-01-98-1778). All procedures were inaccordance with institutional guidelines and were carried out in compliance with the HelsinkiDeclaration. Before the collection of data, all participants completed written informed consent form.

### Measurements of biochemical parameters

Blood samples were collected after 10–12 h fasting and serum and plasma were separated. All of the samples were stored at −80 °C for later biochemical analysis. Fasting serum levels of glucose and lipid profiles, including total cholesterol (TC), triglyceride (TG) and high density lipoprotein (HDL-C) were measured by a Hitachi 912-autoanalyser (Hitachi, Mannheim, Germany) using commercial kits. Low density lipoprotein (LDL) was calculated by friedewald equation. Insulin was measured by radioimmunoassay. Serum irisin concentration was measured using the enzyme-linked immunosorbent assay (ELISA) kits (Biovendor Laboratory Medicine, Modrice, Czech Republic).

### Body composition measurements

After removal shoes and heavy clothing, weight and height were measured using a Seca scale (Seca725 GmbH & Co. Hamburg, Germany), to the nearest 0.1 cm and 0.1 kg, respectively for each participant. BMIwas calculated as weight (in kg) the square of height in meters. Waist circumference was measured by a plastic tape measure locating above the uppermost lateral border of the right ilium, at the end of a normal expiration, to the nearest 0.1 cm in a horizontal plane. Hip circumference was measured by placing the tape around the hips at the biggest circumference of the buttocks using non-stretchable tape measures. Body composition, including total body fat mass and fat-free mass were assessed using Tanitabody composition analyzer (Model TBF-300; Tanita, Tokyo, Japan) during fasting state and before blood sampling. Subjects were required to remove all metal objects, such as earrings, etc., and to wear light clothing before each measurement. The device calculates body fat percentage, fat mass, and fat free mass and predicts muscle mass on the basis of data using BIA.

### Statistical analysis

The normal distribution assumption of continuous variables was assessed using Shapiro-wilk test. Data was expressed as mean ± SD. The analysis ofcovariance (ANCOVA) test was used tocompare mean of irisin level between NWO and controls after adjustment for confounders in threedifferent models. In model I crude association was assessed. In model II energy intake, physical activity, waist circumference (WC) was adjustedand in model III additionally BMI was adjusted. Spearman correlation coefficients were used to determine the relationship between irisin levels and glycemic and lipid profile. All statistical analyses were performed using SPSS version 18 (SPSS Inc., Chicago, IL). The level of significance was considered as ≤0.05.

## Results

Clinical and laboratory findings and demographic characteristics of the study groups are presented in Table 1. The mean age of subjects were participated in this study was 28.95 ± 4.63 years, which was not significantly different between NWO and control groups.

**Table 1:** Baseline anthropometric, clinical and laboratory characteristics of normal weight obese (NWO) subjects and healthy controls

Variables	NWO (*n* = 38)	Controls (*n* = 26)	*P*-value
Age (year)	28.76 ± 4.67	29.23 ± 4.50	0.69
Height (cm)	165.89 ± 4.43	165.32 ± 4.81	0.62
Weight (kg)	62.77 ± 4.77	56.98 ± 4.40	<0.0001
BMI (kg/m^2^)	22.26 ± 1.23	20.88 ± 1.28	<0.0001
Waist circumference (cm)	74.77 ± 4.74	70.84 ± 3.03	<0.0001
Hip circumference (cm)	98.90 ± 4.29	93.44 ± 2.99	<0.0001
WHR	0.75 ± 0.04	0.77 ± 0.03	0.66
Body fat (%)	32.66 ± 2.47	23.79 ± 1.62	<0.0001
Body fat (kg)	20.47 ± 2.71	13.56 ± 1.45	<0.0001
DBP (mmHg)	70.66 ± 13.6	75.50 ± 12.9	0.57
SBP (mmHg)	90.76 ± 12.6	91.5 ± 17.1	0.11
Total cholesterol (mg/dl)	174.00 ± 29.35	174.1 ± 21.44	0.97
LDL- cholesterol (mg/dl)	90.89 ± 18.08	89.53 ± 17.07	0.76
HDL- cholesterol (mg/dl)	59.02 ± 13.70	61.11 ± 9.00	0.46
TG (mg/dl)	87.07 ± 28.28	82.92 ± 26.67	0.55
FBS (mg/dl)	87.71 ± 8.16	84.5 ± 7.3	<0.0001
Insulin (μIU/ml)	9.02 ± 4.75	6.31 ± 2.49	<0.0001

Data are expressed as mean ± SD

*P* < 0.05 is statistically significant

*NWO* normal weight obesity, *BMI* body mass index, *WHR* waist to hip ratio, *DBP* diastolic blood pressure, *SBP* systolic blood pressure, *LDL* low density lipoprotein, *HDL* high-density lipoprotein, *TG* triglyceride

The mean ± SD for weight, waist and hip circumferences and BF% were higher significantly in subjects with NWO compared to controls.

The results showed that thelevelof LDL, TG, FBS and insulin in subjects with NWO were higher than control subjects which was statistically significant for fasting levels of insulin and glucose.

Table 2 shows the crude and adjusted mean levels of irisin in NWO and control groups. In crude model circulating irisin levels were significantly higher in subjects with NWO compared with controls (0.81 ± 0.41*vs* 0.58 ± 0.26 ng/ml, *P* = 0.009). This association remained significant after adjusting for potential confounders in model II (*P* = 0.02) and III (*P* = 0.049).

**Table 2:** Serum level of irisin in subjects with normal weight obesity (NWO) compared to controls

Circulating irisin level (ng/ml)	NWO (*n* = 38)	Controls (*n* = 26)	*P*-value*
Model 1	0.81 ± 0.41	0.58 ± 0.26	0.009
Model 2	0.81 ± 0.36	0.59 ± 0.35	0.02
Model 3	0.82 ± 0.36	0.58 ± 0.40	0.049

Model 1: No adjustment

Model 2: adjusted for energy intake, physical activity, waist circumference

Model 3: adjusted for energy intake, physical activity, waist circumference and BMI

*P-value is for ANCOVA test, *P* < 0.05 is statistically significant

The correlation between circulating irisin levels and biochemical variables are shown in Table 3. In NWO, irisin level was not significantly correlated with all glycemic and lipid profile. In control group, only correlation of irisin with insulin was statistically significant (*P* = 0.03).

**Table 3:** Spearman’s correlation coefficients between serum level of irisin and glycemia/ lipid profile in normal weight obese (NWO) subjects and controls

Variables	NWO (*n* = 38)		Controls (*n* = 26)	
	r	*P*-value*	r	*P*-value*
Total cholesterol (mg/dl)	- 0.02	0.89	0.28	0.15
LDL- cholesterol (mg/dl)	0.001	0.99	−0.23	0.25
HDL- cholesterol (mg/dl)	0.06	0.68	0.05	0.78
TG (mg/dl)	0.11	0.48	−0.06	0.75
FBS (mg/dl)	0.06	0.69	−0.08	0.74
Insulin (μIU/ml)	0.13	0.4	−0.42	0.03

*P-value is for spearman correlation, *P* < 0.05 is statistically significant

## Discussion

The current findings indicated the significantly higher circulating levelof irisinin NWO subjects compared to controls who had normal weight and normal BF%. However, previous trials reported the increased blood level of irisin in the obese subjects. In this context, Saleh et al.found significantly increased irisin level in obese andoverweight women compared to normal weight ones [16].

Similarly, the reports of Ivanov et al. [17], Stengel et al. [10], and Wen et al.[18] showed the positive association between irisin levels and BMI in healthy non-diabetic subjects. The results of Stengle et al. [10] and Huh et al. [7] studies indicated that circulating levels of irisin were higher in healthy subjects with morbid obesity than normal weight controls. On the other hand, Liu et al. reported that circulating irisin level had a negative association with BMI, waist to hip ratio and BF% in men [13]. However in this study, body composition was not measured directly.

It has been suggested that BF% is a better indicator oftotal adiposity compared to BMI. In support of this statement, our results showed that in NWO subject who had normal range of BMI and higher BF%, the serum level of irisin was significantly higher than controls.

Regarding to possible mechanism, it is suggested that increased circulating irisin in obesity is an adaptive compensatory response to obesity-induced disturbed metabolism such as decreased insulin level [16]. Alternatively,” irisin resistance” may be another description for increased levels of irisin in obesity, as has already established for leptin or insulin in obesity [11].

According to our observation in this study, serum irisin levelcorrelated positively with FBS and insulin levels in NWO subjects. This correlation was negative in controls, although it was only significant between irisin and insulin level in controls.

Gomer et al. reported positive correlation between irisin level and HbA1c in T2D patients with and without obesity [19]. Liu et al. showed the positive association between circulating irisin and FBS in non-obese, non-diabetic individuals [13]. In consistence to this results, Huh et al., Stengel et al. and Liu et al. showed positive correlation between serum levels of irisin and FBS [7, 10, 13]. Data from our study showed that the serum levels of FBS and insulin were higher significantly in NWO patients compared to controls. Another study speculated that long time exposure to high blood glucose, irrespective of BMI, was associated significantly with decreased serum level of irisin in diabetic patients [7, 13]. Contrary to type 2 diabetes and despite to higher level of FBS and insulin, NWO patients had higher levels of irisin. Patients with NWO are susceptible to development of T2D; therefore, it is possible that the irisin levels could be decreased in long-time in NWO subjects. In addition, the high serum level of irisin in NWO subjects might be compensatory response to condition called “irisin resistance”, similar to T2 DM.

In agreement with this result, our study showed that the adipose tissue might be the main source of irisin secretion in NWO subjects, because NWO subjects had a higher BF% than controls.

Previous studies found that the activity oftranscriptional co-activator PPAR-γ co-activator-1 α (PGC1α), a molecule up-stream of irisin, in skeletal muscles and therefore circulating irisin level was lower in patients with type 2 diabetes or pre-diabetes than healthy obese subjects [20–22].

On the other hand, in patients with abnormal blood glucose or T2DM, the expressions of FNDC5/irisin are decreased in adipose tissue and skeletal muscles.

Saleh et al. suggested that the glucose intolerance may gradually up regulate the skeletal muscles expression of FNDC5/irisin in non-diabetic subjects [23]. According to this statement and despite to the results of previous studies, we expect the high circulating irisin level in patients with T2DM who are exposed to high level of glucose. To illustrate this conflict observation, we mentioned the results of other studies suggested timelyregulation of local and circulating irisin with tissue-specific mechanisms in different physiological status such as obesity, pre-diabetes and T2DM [24, 25]. Also, Choi et al. and Huh et al. showed that decreased blood irisin level could expose subjects to the development of insulin resistance and T2DM [7, 14]. Similar to the results of Saleh et al., study, we found that serum irisin was significantly negatively associated with insulin level in control subjects [16].

Interestingly, it was shown the opposite expression of FNDC5 in skeletal muscle and adipose tissue of T2D patients compared to lean, obese and pre-diabetic subjects [26]. Diabetic patients displayed the lowest expression of FNDC5 in adipose tissue and circulating irisin level. The muscle expression of FNDC5 was similar in diabetic patients and obese subjects. It is suggested that glucose and lipid, not insulin might be inhibitory regulatory factor in muscle FNDC5 expression in metabolic disorders. It is plausible to think that different interaction exist between expressions of FNDC5 in skeletal muscle and adipose tissue in patients with NWO who had increased insulin and glucose levels. In addition, this expression might change during long term dependant to the glycemic control in NWO subjects.

Our current data also revealed a positive correlation between irisin and HDL-c, LDL-c, TG and a negative correlation association between irisin and TC in NWO subjects. In addition, our results indicate that circulating irisin correlated positively with HDL-c and TCand correlated negatively with LDL-c and TG in control group.

A limited study was performed to evaluate the association between irisin and lipid profile in various populations with obesity or diabetes and both of them. Liu et al. reported that serum level of irisin correlated positively with total cholesterol and TG in non- obese, non- diabetic subjects [13]. The significant association between irisin and HDL-c in healthy non-diabetic subjects in Benton et al. study, suggested a protective role for circulating irisin in cardiovascular disease [27], which is similar to our result in NWO and controls. Another study conducted by Sanchis-Gomer et al., reveals no significant relationship between irisin and TC, TG and LDL [28]. In contrary, Wen et al. study showed a significantpositive correlations of TC, TG and LDL with circulating irisin in non- diabetic participants [18]. The positive association between serum level of irisin and TG was shown in Liu et al. study [13]. Previous studies showed that muscle FNDC5 was positively associated with TG [7], and negatively associated with HDL-c [7, 12]. For the first time, Saleh et al. reported that serum irisin has a positive relationship with both leptin and insulin in type 2 diabetes [16]. It is plausible to think that the association between irisin and lipid profile may be mediated by its effect on leptin.

The correlation of irisin with TG, LDL-c and HDL-c was inversely between NWO and control subjects. More studies are needed to establish the detail association between irisin and lipid profiles in subjects with NWO compared to obese, diabetes and non- obese, non-diabetic healthy subjects. In addition to skeletal muscles and adipose tissue, FNDC5 mRNA/irisin detected in various tissues, including pericardium, kidney, liver, lung and neurons in human [7, 9, 29–31]. It was suggested thatthe muscle/adipose irisin secretion ratio might vary dependent to the physiological status. In athletes with trained muscles by exercise, muscle tissue would actively increase FNDC5/irisin but, in obesity, the adipose tissue is the main source of FNDC5/ irisin secretion [32].

The limitations of our study are its small sample size and lack of information about the FNDC5 expression and irisin level in adipose tissue, skeletal muscles and its ratio in subjects with normal weight obesity. There was no data about the life quality and socioeconomic factors in our study that considered as a limitation of our study. The strength of our study is that this is the first study that investigated blood irisin level and its association with biochemical parameters in subjects with normal weight obesity.

## Conclusion

In summary, the results of this study demonstratedserum irisin levels were higher in NWO subjects than controls. In control group, only the negative association between irisin and insulin levels was statistically significant. Further studies with larger sample size are clearly needed to evaluate the potential role of irisin in NWO subject and other disturbed metabolic conditions.

## Abbreviation

BAT, brown adipose tissue; BIA, bio electrical impedance analysis; BMI, body mass index; FBS, fasting blood sugar; FFM, free fat mass; FM, fat mass; FNDC5, fibronectin type 111 domain 5; HDL, high density lipoprotein; LDL, low density lipoprotein; NOW, normal weight obesity; PGC1 α, PPARγ-coactivator-1α; REE, resting energy expenditure; UCP, uncoupling proteins; WAT, wight adipose tissue; WHR, waist to hip ratio
